# LIPSS Applied to Wide Bandgap Semiconductors and Dielectrics: Assessment and Future Perspectives

**DOI:** 10.3390/ma15041378

**Published:** 2022-02-13

**Authors:** Matteo Mastellone, Maria Lucia Pace, Mariangela Curcio, Nicola Caggiano, Angela De Bonis, Roberto Teghil, Patrizia Dolce, Donato Mollica, Stefano Orlando, Antonio Santagata, Valerio Serpente, Alessandro Bellucci, Marco Girolami, Riccardo Polini, Daniele Maria Trucchi

**Affiliations:** 1 ISM-CNR, DiaTHEMA Laboratory, U.O.S. Montelibretti, Via Salaria km 29.300, 00015 Monterotondo, Italy; matteo.mastellone@ism.cnr.it (M.M.); valerio.serpente@ism.cnr.it (V.S.); alessandro.bellucci@ism.cnr.it (A.B.); marco.girolami@ism.cnr.it (M.G.); polini@uniroma2.it (R.P.); daniele.trucchi@ism.cnr.it (D.M.T.); 2 ISM-CNR, FemtoLAB, U.O.S. Tito Scalo, Zona Industriale, 85050 Potenza, Italy; marialucia.pace@tiscali.it (M.L.P.); patrizia.dolce@ism.cnr.it (P.D.); donato.mollica@ism.cnr.it (D.M.); stefano.orlando@ism.cnr.it (S.O.); 3 Dipartimento di Scienze, Università della Basilicata, Viale dell’Ateneo Lucano 10, 85100 Potenza, Italy; mariangela.curcio@unibas.it (M.C.); nicola.caggiano002@studenti.unibas.it (N.C.); angela.debonis@unibas.it (A.D.B.); roberto.teghil@unibas.it (R.T.); 4 Dipartimento di Scienze e Tecnologie Chimiche, Università di Roma ‘Tor Vergata’, 00133 Rome, Italy

**Keywords:** LIPSS, wide bandgap semiconductors, dielectrics, surface nanostructuring, LSFL, HSFL, SSPs

## Abstract

With the aim of presenting the processes governing the Laser-Induced Periodic Surface Structures (LIPSS), its main theoretical models have been reported. More emphasis is given to those suitable for clarifying the experimental structures observed on the surface of wide bandgap semiconductors (WBS) and dielectric materials. The role played by radiation surface electromagnetic waves as well as Surface Plasmon Polaritons in determining both Low and High Spatial Frequency LIPSS is briefly discussed, together with some experimental evidence. Non-conventional techniques for LIPSS formation are concisely introduced to point out the high technical possibility of enhancing the homogeneity of surface structures as well as tuning the electronic properties driven by point defects induced in WBS. Among these, double- or multiple-fs-pulse irradiations are shown to be suitable for providing further insight into the LIPSS process together with fine control on the formed surface structures. Modifications occurring by LIPSS on surfaces of WBS and dielectrics display high potentialities for their cross-cutting technological features and wide applications in which the main surface and electronic properties can be engineered. By these assessments, the employment of such nanostructured materials in innovative devices could be envisaged.

## 1. Introduction

Theodore Harold Maiman, with the first solid-state ruby laser discovered in 1960, illustrated the large potentialities of his invention through the statement “*a solution looking for a problem*”. Since then, lasers have been applied successfully in different industrial and manufacturing processes such as, for instance, welding, material treatments and cutting. During the 1980s, materials science saw a boost in employing pulsed lasers for thin-film depositions by the so-called pulsed laser ablation and deposition (PLD) technique employed for a wide range of innovative materials such as superconductors, semiconductors, ceramics and alloys, with well-defined compositions, phases and properties [1,2,3,4,5,6,7,8].

During the last two decades, the laser ablation process has gained, day by day, significant importance in developing new materials by using different pulse durations ranging from ns to fs time scales. Both ns and fs pulses have shown great versatility in providing materials suitable for numerous applications, for instance, being bioactive for osseointegration prosthesis, catalysts, the conversion or storage of energy and plasmonic, electronic, thermionic, thermoelectric and photonic devices. Most of these can be obtained in different environmental conditions such as in vacuum, inert or reactive background gas as well as in liquid [9,10,11,12,13,14,15,16,17,18]. Laser ablation performed in liquid has opened new opportunities for obtaining, straightforwardly, very stable colloidal nanoparticles suspensions without the use of either reactive chemicals or stabilizers. 

With the widespread availability of highly powerful ultrashort laser pulses, in the range of an fs-ps time scale, even the pulsed laser deposition technique has been demonstrated to be suitable for obtaining nanostructured thin-film systems, mostly made of nanoparticles retaining, in general, the starting ablated target material composition [1,2,3,4]. Nevertheless, to trigger such processes, an ablation threshold should be provided with densities of energies per surface unit (fluences) in the order of 1–10 J/cm^2^, which, in any case, is dependent on both the kind of materials and the laser properties used. When certain conditions are fulfilled at laser fluences close to the ablation/damage threshold, however, the laser–matter interaction can induce the formation of nano- and micro-patterned surfaces defined, in general, as Laser-Induced Periodic Surface Structures (LIPSS). The features of these are strongly dependent on the laser beam parameters (wavelength, pulse duration, fluence, angle of incidence, repetition rate, number of spatial effective incident pulses, etc.) as well as the material physical-chemical features. In this regard, different process routes described by suitable models, or a combination of them, can be considered. Even though laser pulses lasting longer than fs-ps timescales, like the pioneering work by Birnbaum published in 1965 [19], can provide materials’ surface patterning, ultra-short (ps-fs) pulsed lasers are almost exclusively employed with this purpose for their ability to limit the thermal effects of the laser–matter interaction process. 

Ultra-short pulsed lasers are effective in providing the formation of periodic structures on the surface of any kind of material (metals, semiconductors and dielectrics). These structures can display spatial periods, *Λ*, characterized by ripples (*Λ* ≤ *λ*) or grooves (*Λ* > *λ*), where *λ* represents the wavelength of the incident pulsed laser beam. When ripples display periods in the range of (*λ*/2 < *Λ* ≤ *λ*), near-subwavelength ripples “NSRs” or, more generally, a Low Spatial Frequency LIPSS (LSFL) regime is considered; on the contrary, if *Λ* ≤ *λ*/2, the periodic structure is defined as High Spatial Frequency LIPSS (HSFL). It is important to highlight that the current literature is inconsistent regarding the definition of LSFL and HSFL in terms of the periodicity/wavelength ratio, and in this work we decided to adopt the definition provided by Bonse et al. in Refs. [20,21]. In addition, HSFL can even be defined as deep-subwavelength ripples “DSRs” when *Λ*/*λ* ≤ 0.4 [22]. Regarding the alignment, for any material, when the two spatial periods LSFL or HSFL take place, these might be parallelly or normally oriented to the linear polarization of the incident laser pulse.

Apart from the progress achieved in scaling up the process at large area rates (in the order of m^2^/s), the direct capability of using pulsed laser beams in structuring material surfaces in spatial domains much lower than the optical diffraction limit, that is HSFL, represents a huge benefit for potential industrial applications of LIPSS. In this scenario, LIPSS of wide bandgap materials and dielectrics could add even more perspectives for the employment of this technique. In this regard, the modification of electronic features such as, for instance, the generation of intermediate states within the bandgap can be obtained, and a new preparation method for optoelectronic components could be provided. 

## 2. LIPSS Models’ Overview

LIPSS’ surface topography modulation depends on both the processing parameters used and the electronic properties of the treated materials. LIPSS of wide bandgap semiconductors (WBS) or dielectrics is often characterized by *Λ* ≈ *λ*/*n*, where *n* refers to the material’s refractive index. For highly absorbing materials, such as metals and low bandgap semiconductors, *Λ* is generally comparable to *λ*. A comprehensive review on the different models developed on LIPSS has been published by Bonse et al. [21], which, for its completeness, represents a useful guide for readers who require an in-depth survey of the possible approaches to the phenomena occurring in laser-induced surface structures. 

In Ref. [21] and references therein, the contributions played by the electromagnetic and matter reorganization models are well evidenced, which take place at timescales in the range of fs-ps and ps-μs, respectively. The main challenge in modelling LIPSS is related to the phenomenon complexity, due to the combination of processes occurring at various timescales and spatial constraints spanning from atom dimensions up to the affected volume of the patterned material. Thus, for metals, Finite-Difference Time Domain (FDTD) calculations have recently been employed in combination with material-reorganization approaches. These are based on the Two-Temperature Model (TTM) and either the material response at the atomistic level by Molecular Dynamic (MD) simulations or material phases involved by the Navier-Stokes Equation (NSE) to take into account material displacements occurring in the laser-induced melted pools [23,24,25]. 

Regarding LIPSS of wide bandgap materials and dielectrics, i.e., Vis-IR weakly absorbing material, processes occur in the fs-ps timescale domain, and the electromagnetic models should be considered. In these circumstances, several kinds of electromagnetic waves can interfere with the oncoming pulsed laser beam, which can spatially modulate its absorption on the material’s surface, such as scattered radiation due to the starting roughness of the material [26,27] and surface polaritons’ excited modes. Among these, Surface Plasmon Polaritons (SPPs) [28,29,30] are of particular interest.

SPPs are generated by coherent electron densities bound and oscillating at the interface of the two involved media (e.g., air and laser irradiated material’s surface). SPPs excitation is allowed only after precise conditions are met regarding the coupling of light with plasmonic active interfaces. In general, plasmonic effects are modelled for noble metals, whereas for other materials, a refinement of the common SPPs theory is needed to determine the occurrence of plasmon-active interfaces [31]. When ultrashort laser pulses are employed, a high density of electrons, *N_e_*, are excited in the conduction band above a critical value. In these circumstances, even initially plasmonically nonactive materials, such as WBS and dielectrics, can act as metals, behaving, transiently, as SPPs-active materials [22,32]. The effectiveness of the transient SPPs-active behavior of such materials is dependent on the cross-sections of nonlinear multiphoton processes exciting electrons to the conduction band. Since multiple laser pulses can change the surface roughness and consequently its capability to absorb the impinging laser beam, it follows that accumulated pulses can affect the spatial period of the forming ripples. To consider the role played by multiple laser pulses, a Drude model has been used to evaluate the dielectric permittivity as a function of the number of induced excited carries in the conduction band by successive laser pulses [22,33]. 

Relevant in modelling the formation of LIPSS is the work by Sipe et al. (Sipe’s theory), which, starting from Maxwell’s equations, relates the dielectric polarization density to the material surface roughness, including (1) the excitations of the SSPs induced and (2) the interference of SSPs with the incident pulsed laser beam [27,34,35]. In this manner, the laser-induced structured periodicity can be associated with both the laser and materials’ surface parameters, which are, for instance, the wavelength, angle of incidence, polarization direction, surface roughness, and dielectric permittivity, *ε*, respectively. Sipe’s theory accurately predicts the LSFL of plasmonically nonactive and active materials, which are a wide bandgap, dielectric, and metals, respectively. For the former, LSFL periodicity (*Λ* ≈ *λ*/*n*) and orientation (parallel to the linear laser polarization) are foreseen where radiation remnant electromagnetic field structures dominate on radiation fields and surface polaritons. The radiation remnants originate from specific non-propagating electromagnetic modes close to the rough surface. Sipe’s theory, however, does not consider changes occurring during the laser-matter interaction process, which can vary the material’s roughness and dielectric permittivity. The latter must be considered for wide-bandgap materials and dielectrics where multiphoton carriers’ excitation processes are involved at the high laser intensities used. These affect the material transient metallic behavior and relative SPPs induced during the laser pulse itself so that their effects increase, whereas radiation remnants’ role decreases. 

To solve this inter-pulse feedback, namely the changes of dielectric permittivity occurring when a critical density of excited electrons in the conduction band is attained during the laser pulse incidence, Sipe’s theory has been combined with Drude’s model (Sipe–Drude model) [32,36,37,38,39,40,41,42]. Accounting even for the in-depth propagation and effects governing the incident laser pulsed beams’ distribution, that is underneath the material surface, Finite-Difference Time-Domain (FDTD) simulations are used. These allow one to solve Maxwell’s equations at even deeper-lying sub-surface regions of various topography surfaces [43,44,45,46]. These confirm the validity of Sipe–Drude simulations, providing qualitatively, in the Fourier space, the LIPSS fingerprint of light scattering and localized absorption, so that *Λ* ≈ *λ*/2*n* HSFL, named type-r nanostructures, can occur in WBS and dielectric materials [45,46].

Skolski et al. introduced, for their FDTD approach, the inter-pulse feedback where the effect caused by material removal through ablation is considered [47]. Buschlinger et al. [48] show, via the FDTD approach, how the plasma generation induced by multiphoton absorption of intense pulsed laser beams in dielectrics is strongly amplified around nanometer-sized inhomogeneities. These plasma structures, due to randomly distributed inhomogeneities, interact strongly, organizing themselves in regularly spaced planes oriented perpendicularly to the laser polarization. These can justify HSFL nanogratings obtained in fused silica and tellurium dioxide and displaying periodicities of about 20–30 nm, respectively [49,50]. Nanograting and type-r nanostructures can both occur during the laser pulse incidence due to their origin induced by the interference between the laser beam electromagnetic field and the nonradiative near-field, which are scattered, coherently, at the surface’s nanodefects [44]. Such behavior enhances, locally, the laser absorption and the generated electromagnetic fields and scattering. However, when more repetitive laser irradiations are impinging on the material surface, so that, consequently, laser pulses’ incubation effects take place, the HSFL turns into the sub-surface induced LSFL with periodicity *Λ* ≈ *λ*/*n.*

During the time duration of ultrashort laser pulses, the absorption of radiation, occurring through electron excitations of the material, is not able to be transferred to the lattice by heat diffusion. Before thermalization occurs, the Two-Temperature Model (TTM) is considered, where electrons and lattice/ion temperatures are not in equilibrium between them. Electron–phonon coupling occurs, depending on the material involved, in the range of 0.1–10 ps, which is often too short to act in tandem with the oncoming laser beam. In silicon, the thermal effects do not cancel the free carrier density oscillations after 1 ps delay modulating the lattice temperature profile. During laser inter-pulse ablation, melting, material displacement and solidification are induced and LIPSS formation follows [51]. In this timescale scenario, even structural and chemical changes can occur on the material’s surface. For dielectrics, such as fused silica, supra-wavelength periodic surface structures following melting and subsequent capillary and reorganizing effects have been observed after the incidence of ultrashort laser pulses [52]. To deal with the main and general outlines and open questions about LIPSS for such materials, it is suggested to refer to a recent explicative perspective paper by Bonse and Gräf [53].

Although LIPSS formation theoretical models represent the basis for grasping the mechanisms involved during laser–matter interaction processes, one further consideration must be introduced regarding the materials’ laser-induced electronic structured features. In this framework, as it will be shown in the next section, the use of Density Functional Theory (DFT) can provide, in its first approximation, evidence about the optical properties following the fabrication of LIPSS on WBS surfaces. 

## 3. Surface Modifications of WBS and Dielectrics

**Figure 1:**
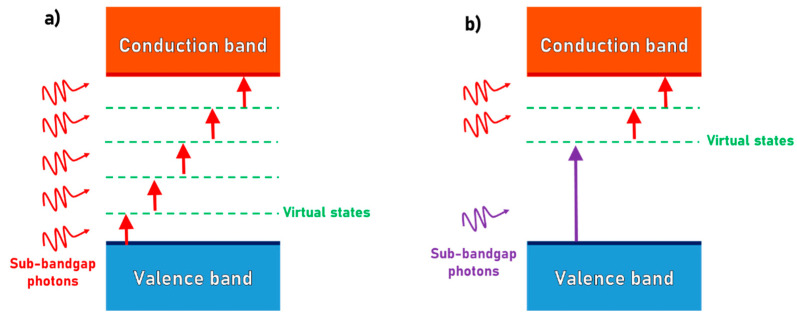
Schematic depiction of (**a**) multiphoton absorption of sub-bandgap photons and (**b**) multiphoton absorption of dual-wavelength double-pulse irradiation.

As previously described, ultrashort (fs-ps) laser pulses can generate nano- and micro-patterning on the surfaces of WBS and dielectrics despite the fact that these materials often have extremely high laser ablation thresholds and are transparent to most operating wavelengths (i.e., Vis-IR) of commercially available laser systems. The ultrashort laser beams have the capability of triggering, with increased efficiency, non-linear processes such as multiphoton absorption and tunnelling ionization involving virtual states [54]. Both effects promote the excitation of electrons from the valence band to the conduction band by multiple absorptions of sub-bandgap photons (Figure 1a). These band-to-band electronic excitations occur in WBS, such as silicon carbide (SiC), diamond and zinc oxide (ZnO), as well as dielectrics, such as glasses and ceramics. Moreover, recent studies have demonstrated that multi-photon absorption can be further enhanced by employing dual-wavelength double-pulse irradiation [55], allowing efficient micromachining processing for materials with bandgaps (*E_g_*) up to about 10 eV, such as sapphire (α-Al_2_O_3_). 

Using dual-wavelength double-pulsed lasers (see Figure 1b), the efficiency of the laser-induced excitation of electrons into the materials’ conduction band can be enhanced by lowering the number of photons involved and choosing a proper delay time between the two different laser beams. These parameters can play a role in providing the formation of nanostructures with high accuracy as they do in laser machining induced in sapphire wafers [55]. The process that drives electrons from the valence band to the conduction band is regulated by the inter-pulse delay time, which must be shorter than the lifetime of electrons excited on the virtual or laser-induced inter-band localized states. The latter, which are transient states, can be due to the formation of the point lattice’s defects, such as color centers [55], which can be exploited for new developments of LIPSS in WBS.

The irradiation of the material surface by ultrashort pulses provides another distinctive advantage: the confinement of the photon absorption solely to the focal volume [56]. This effect offers a more precise material processing by minimizing undesired thermal effects such as structural micro-cracks, pits, craters or melted regions.

Among the many nano- and micro-processing techniques that exploit the multiphoton absorption in WBS and dielectric materials, the development of LIPSS represents a new cutting-edge technique. Indeed, the surface engineering through fs-laser irradiation is used to modify and functionalize the surface properties of WBS and dielectrics without employing invasive and time-consuming methods, such as photolithography or chemical processing. This topic is rapidly gaining attention from both the industrial and scientific communities, thanks to the remarkable physical and chemical properties of these materials. WBS meet the strict criteria that the development of high-power, high-temperature electronic devices require, such as high breakdown fields, low dielectric constants, extremely high thermal conductivities and inertness to most chemical reagents, even at high temperatures (≥ 600 °C). Similarly, ceramics offer characteristics that make them particularly suitable for being employed in applications operating in harsh environments. Among their properties, it is important to mention high hardness, strength and melting points, low emittance at high temperatures and consequently good spectral selectivity. Not unlike the aforementioned materials, transparent dielectrics, such as fused silica, are also rapidly attracting interest since nano- and micro-patterning can extend the field of their potential applications, especially in optics-related fields. 

During the last two decades, many WBS and dielectrics were successfully nano- or micro-patterned using ultrashort laser pulses. The pioneering work by Höhm et al. [36,57] on fused silica and by Dufft et al. [37] on ZnO allowed to clarify several aspects of the formation mechanism of both LSFL and HSFL on these classes of materials. Their studies confirmed that the origin of LSFL is correlated with an inhomogeneous energy deposition, caused by the interference of the impinging radiation with surface-scattered waves followed by consequent localized ablation. Moreover, according to Dufft et al., the induction of HSFL on WBS can be ascribed to the generation of frequency-doubled radiation, also known as second harmonic generation (SHG) [37], onto the material surface, pointing out, additionally, that the initial surface roughening and the near-surface material modification could greatly enhance the scattered SHG at the material’s surface. This theory indicates that, as anticipated in Section 2, the spatial period of LIPSS, *Λ*, is correlated with the incident laser wavelength, *λ*, and the material refraction index, *n*, according to the relationship:

*Λ* = *λ*/2*n*. (1)

**Figure 2:**
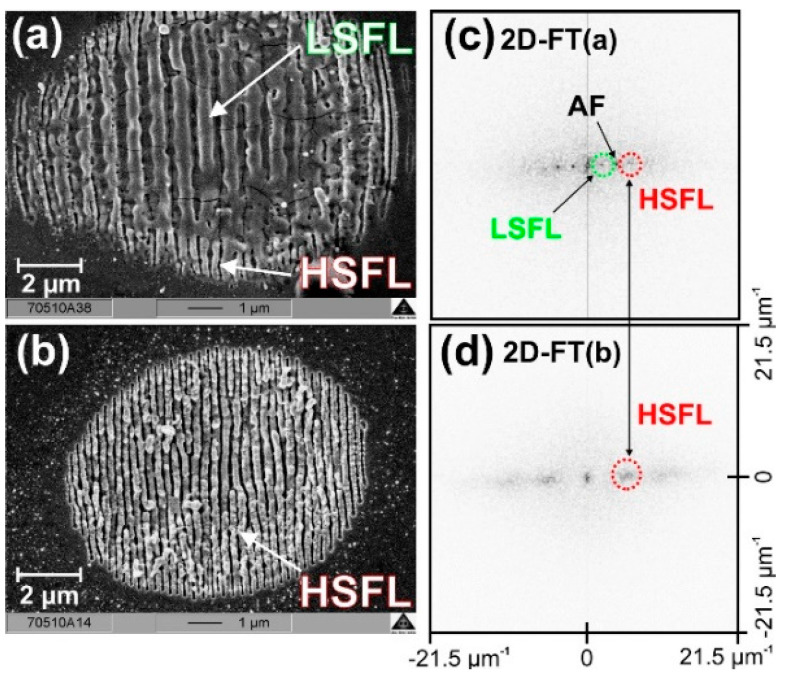
(**a**) SEM micrographs of ZnO after irradiation with 10 laser pulses at a fluence of 0.62 J/cm^2^; (**b**) SEM micrographs of ZnO after irradiation with 50 laser pulses at a fluence of 0.48 J/cm^2^; (**c**) 2D−FFT of the SEM micrograph (**a**); (**d**) 2D−FFT of the SEM micrograph (**b**); AF indicates an artifact of the 2D-FFT process of 2c. Reproduced with permission from Ref. [37]. Copyright 2006 American Institute of Physics.

In their experiment, Dufft et al. irradiated ZnO (*E_g_* = 3.37 eV) with linearly polarized laser pulses at a wavelength of 800 nm and a pulse duration, *τ,* of 200 fs. The authors were able to obtain both LSFL (*Λ* ≈ 680 nm) and HSFL (*Λ* ≈ 230 nm) as it is shown in Figure 2 (reproduced from Ref. [37]). Both structures resulted in being perpendicular to the laser polarization and were originated by different conditions, which are the number of pulses and fluences, with LSFL being fabricated at higher values of the latter.

**Figure 3:**
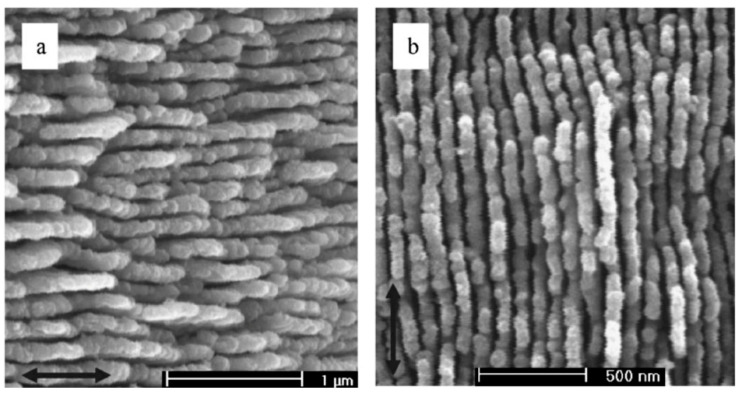
SEM micrographs of 6H-SiC after pulsed laser irradiation. (**a**) Surface morphology after irradiation with 500 consecutive pulses at 800 nm. (**b**) Surface morphology after irradiation with 500 consecutive pulses at 400 nm. Reproduced with permission from Ref. [58]. Copyright 2006 American Institute of Physics.

The validity of Equation (1) was demonstrated by employing different wavelengths on 6H-SiC (*E_g_* = 3.0 eV), as reported in the work of Jia et al. [58], where the material was irradiated with linearly polarized laser pulses at wavelengths of 800 and 400 nm, and a pulse duration, *τ*, of 130 fs. These gave rise to HSFL with *Λ* equal to 150 and 80 nm, respectively (see Figure 3).

In Refs. [37,58] experiments, emission peaks at half of the wavelength of the incident laser pulses were observed, suggesting SHG as the main mechanism giving rise to HSFL. As already mentioned in Section 2, FDTD studies suggest that the periodic structures originate from the coherent superposition between the scattered near-field at the surface and the incident electromagnetic field produced by the propagating beam [43,44]. This work led to a shift in focus on the field, considering the possibility of tailoring advantageous modifications of different properties for these materials and showing, as well, that (a) the explanation of the occurring phenomena needs to be evaluated carefully for each condition, and (b) the general models have to be considered with caution. 

Among WBS, diamond represents one of the most-studied materials for its remarkable electronic, thermal and surface properties as well as its suitability for technological cutting-edge applications. The fabrication of LIPSS on diamond (*E_g_* = 5.5 eV) was first reported in 1999 in the work of Ozkan et al. [59] in which a linearly polarized laser wavelength of 248 nm and a pulse duration of 380 fs were used. The authors showed the formation of ripple structures with spacing in the order of the laser wavelength but with poor homogeneity. In the following years, both the possibility of extending the nano-patterning to large-area surfaces and the concurrent modification of diamond optical and electronic properties were demonstrated. In 2014, Calvani et al. proved that an ordered, one-dimensional patterning with a periodicity defined by Equation (1) (*Λ* ≈ 170 nm by 800 nm fs pulsed laser, linearly polarized, *τ* = 100 fs) was able to drastically increase the absorptance in the Vis-IR spectral region. The overall solar absorptance, *α_solar_*, increased from 20% up to values of 85% without perturbing the film’s electronic and transport properties [60,61]. The solar absorptance is defined as the integral in the solar wavelength range (300–3000 nm) of the absorptance *A(λ)* multiplied by the solar irradiance *W_solar_*(*λ*), divided by the integral of the solar irradiance itself, that is:

(2) $$\alpha_{solar}=\frac{\int_{\lambda_{1}}^{\lambda_{2}}A\left(\lambda \right)W_{solar}\left(\lambda \right)d\lambda }{\int_{\lambda_{1}}^{\lambda_{2}}W_{solar}\left(\lambda \right)d\lambda }.$$

where *W_solar_*(*λ*) is the global-tilt GT 1.5 airmass AM solar irradiance in the wavelength range between *λ*_1_ = 300 nm and *λ*_2_ = 3000 nm [62]. This parameter provides quantitative information on the overall absorption of the solar radiation by the material. The increase in the absorptance can be attributed, at a glance, to the light-trapping action accomplished by the regular periodic nanostructures, but also to the heightening of the absorbing area and surface roughness induced by the formation of superficial structures. All these aspects contribute to the enhancement of the interaction between the surface and impinging radiation. Surface texturing is, however, not only optically but also electronically active. The simple geometrical variation of the surface at the nanoscale is demonstrated to induce a change in the resistance [63] and Fermi level [64] in semiconductors, called geometry doping. Moreover, additional defect levels can be produced within the semiconductor bandgap, acting, effectively, as an intermediate band of defect states. In this regard, DFT calculations have predicted that defected CVD diamond presents strong photon absorption in the near-infrared and visible region by the formation of intermediate bands within the material’s bandgap, which is related to laser-induced intrinsic defects. These, in fact, can exhibit diamond’s sub-band gap absorptions, which are in turn strong and weak at less than 0.7 eV and in the range of 2.2–3.1 eV, respectively. Vacancies are tuned by the laser doses whose LIPSS periodicity is also related to [65]. Furthermore, DFT calculations may offer other perspectives for LIPSS applications such as the opportunity to use complex materials such as diamond-based compounds [66], or n-type AlN doped by silicon as a consequence of SiH_4_ gas phase decomposition in the vicinity of an AlN surface or other point defects [67]. By these theoretical calculations and their quasi-particles corrected gaps, even new types of materials could be considered as WBS for LIPSS applications such as transition metals encapsulated in silicon cages clusters (MSi_12_, MSi_16_) displaying, in the gas phase, energy gaps in the range of 2.45–5.64 eV. These, might represent nanotemplates or building blocks of assembled phases for new applications of WBS for optoelectronic nanodevices [68]. In order to determine the excited states of all systems, Time-Dependent DFT (TDDFT) approaches could be applied, even though, because of likely nonlinear processes involved, it would be more appropriate to employ methods based on Green’s functions. 

The large photoelectronic boost in diamond was demonstrated in Ref. [60], with the achievement of a significant increase of two orders of magnitude in the sub-bandgap quantum efficiency (i.e., the efficiency in transforming absorbed photons into exploitable electrons). These remarkable results demonstrated how surface nano-texturing provided diamond the newfound capability in absorbing solar radiation opening the path for its use in thermal solar applications, as a simple selective absorber or, more ambitiously, as the active material in the development of solar cells working at high temperatures.

**Figure 4:**
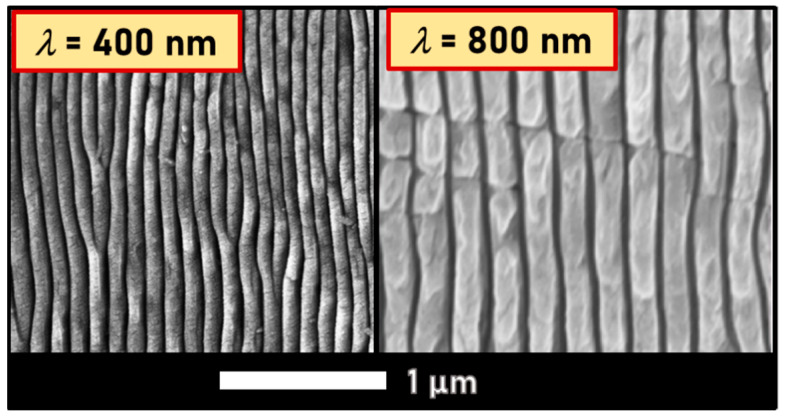
Comparison between SEM images of HSFL obtained with different laser wavelengths on polycrystalline diamonds samples. Linearly polarized pulses, *λ* = 800 nm, *τ* = 100 fs.

Subsequent studies confirmed that HSFL on diamond (similarly to SiC) is generated with periodicities that are strictly connected to the incident laser beam wavelength. By decreasing *λ* to 400 nm, Girolami et al. were able to fabricate LIPPS with a periodicity of approximately 80 nm [69], thus confirming the expected value of *λ*/2*n*, (*n* = 2.46 for diamond at the wavelength of 400 nm), as it is shown in Figure 4. The experimentally validated relationship suggests that it is possible to finely modulate the texturing periodicity of diamond by selecting the proper laser wavelength to be used. 

Diamond nanostructuring can be useful not only for solar applications, but also for other kinds of devices. In 2017, Granados et al. [70] reported the capability of Boron-Doped Diamond (BDD) to become highly hydrophilic after nanostructuring its surface, whereas Sartori et al. [71] demonstrated that surface nano-patterning is a cleanroom-free and flexible processing technique for adjusting the electro-chemical properties of BDD. Both works aimed at developing electrodes for (bio)sensing, catalysis or energy storage. Additionally, in 2020, Amoruso et al. [72] showed that the same technique allows one to tailor the absorption selectivity for polarized THz radiation in the range of (0.25–3) THz, proving the extreme versatility of surface nano- and micro-patterning. In that work, another aspect of surface functionalization was evidenced, as the surface was not only nano-patterned, but also graphitized, leaving a conductive overlayer on the surface able to modify the response of the system to THz radiations. Indeed, the interaction of the ultra-short-pulsed laser with diamond can also cause the graphitization of the material when the laser fluence is carefully tuned to locally induce avalanche photoionization. This phenomenon occurs when the already promoted electrons present in the conduction band are excited to even higher energy states because of the absorption of multiple incoming laser photons. By acquiring a high enough kinetic energy, the promoted electrons can excite other bound electrons giving rise to the avalanche effect [56]. The graphitization of diamond can also be extended within the bulk to create conductive micro-paths [73] acting as distributed electrodes for electronic applications. Recently, it was demonstrated that it was also possible to develop LIPSS even on other highly technological carbon-based materials such as graphene [74].

Another WBS of large interest is SiC, which is currently successfully utilized in high-power and high-frequency electronics and micro-electro-mechanical systems (MEMS) [75,76,77]. In 2006, Jia et al. [58] were the first to report the fabrication on 6H-SiC of LIPSS with subwavelength periodicity using the laser wavelengths, λ, of 800 and 400 nm and with a pulse duration, *τ*, of 130 fs. Subsequently, Zhao et al. [78] carried out the first attempt aimed at tailoring the optical properties of n-doped 6H-SiC, obtaining a 39% enhancement of the total absorption integrated from 1.25 to 3.0 eV. Song et al. [79] showed the possibility to finely tune the birefringence properties of the treated surfaces by controlling both the single-laser pulse energy and scanning velocity of the sample, that is, varying the accumulation of laser pulses on the material’s surface.

In 2020, Mastellone et al., [80] working on semi-insulating 6H-SiC (linearly polarized pulses, *λ* = 800 nm, *τ* = 100 fs), further enhanced the optical absorption of the material reporting an increase in the overall solar absorptance, *α_solar_*, from 10% up to values of 75%. Furthermore, the spectral selectivity at 1000 K, which is evaluated through the absorptance/emissivity (*α*/*ε*) ratio, increased from 0.3 (pristine sample) to approximately 1.7, which is a remarkable achievement when compared with materials specifically developed for solar applications (*α/ε* > 1.5) [81,82].

**Figure 5:**
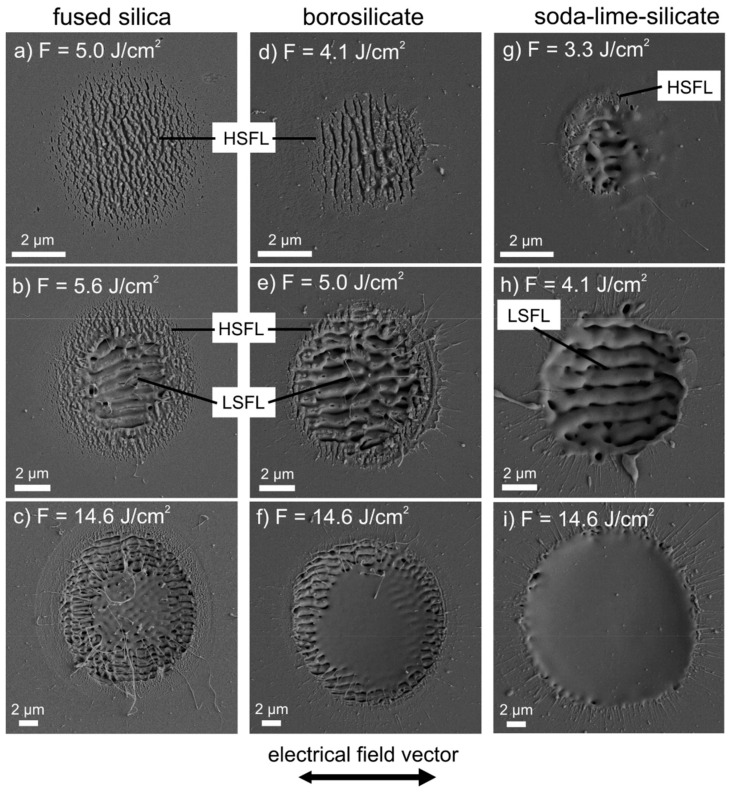
SEM micrographs of fused silica (**a**–**c**), borisilicate glass (**d**–**f**) and soda-lime-silicate glass (**g**–**i**) after irradiation with five laser pulses at different peak fluences. Reproduced with permission from Ref. [42]. Copyright 2017 under Creative Commons BY 4.0 license.

LIPSS can also be fabricated on technically relevant glasses. In 2017, Gräf et al. [42] proved that by using laser pulses at a wavelength of 1025 nm and a pulse duration of 300 fs, both HSFL and LSFL on fused silica (E_g_ up to 9 eV), borisilicates and soda-lime-silicates were induced. LIPSS morphology and alignment (orthogonal to the polarization of the laser irradiation) were similar for the investigated materials. Visual depictions of the surface nanostructuring are reported in Figure 5 (reproduced from Ref. [42]). As it was for ZnO, the authors demonstrated that both LSFL (600 ≤ *Λ* ≤ 1000 nm) and HSFL (not reported in the paper) can be imprinted on the different classes of technical glass and that, yet again, LSFL forms at higher peak fluences.

**Figure 6:**
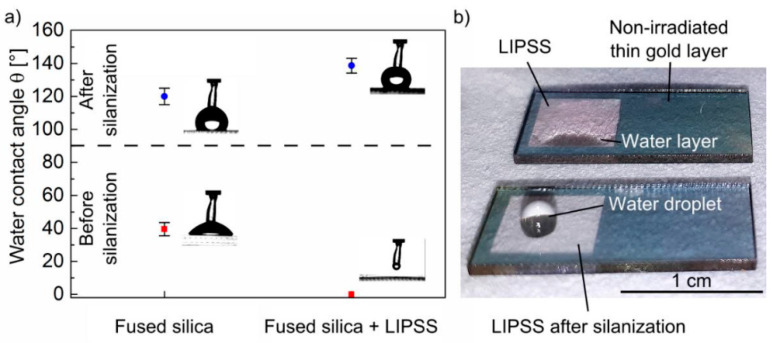
Contact angle analysis of pristine and LIPSS fused silica before and after SiH_4_ functionalization. (**a**) Water contact angles before and after silanization for pristine and LIPSS fused silica; (**b**) Photographs of water droplets onto LIPSS fused silica before (top) and after (down) silanization. Reproduced with permission from Ref. [83]. Copyright 2020 under Creative Commons BY 4.0 license.

Recently, Kunz et al. [83] have shown that regular large-area nanostructuring was attainable on fused silica using an innovative technique that was focused on irradiating a sample, pre-coated by a thin gold layer, with an fs-laser operating at λ = 1025 nm and τ = 300 fs. The coating layer, which was completely ablated during irradiation, enabled the reproducible generation of homogeneous LIPSS by reducing the impact of material properties and laser energy fluctuations on the process. Furthermore, in the same work, the authors report how the change in morphology combined with a functionalization of the surface by SiH_4_ changed the wettability properties of the material as it occurs with the LIPSS of metals [84]. As it is possible to see in Figure 6 (reproduced from Ref. [83]), the silanization of the nano-structured surface is able to completely vary the LIPSS material’s wettability, transforming its behavior from hydrophilic to superhydrophobic.

Similar experiments conducted on sapphire at a wavelength of 1030 nm and with a pulse duration of 222 fs produced LSFL with an orientation orthogonal to the laser polarization and a periodicity of 924 ± 6 nm [85], in accordance with the results obtained on technical glasses.

Lastly, it is important to mention that the nano- and micro-patterning of surfaces is also viable on uneven and/or defective surfaces, typical of ceramic composites. Ultra-high temperature ceramics (UHTCs) have been recently proposed as innovative, high-temperature, thermal solar absorbers due to their unique thermal stability and intrinsic spectral selectivity. In this context, surface texturing at the nanoscale was introduced in the past decade with the aim of improving the performance of selective solar absorption. In a similar fashion to WBS, LIPSS with a periodicity between 300 and 800 nm provided enhancement of the solar absorptance value for many ceramic composites. For instance, it was demonstrated that the absorption spectra of a sintered complex ceramic material, hafnium carbide, stabilized with molybdenum disilicide (5% volume), could be increased by surface nano-texturing [86]. Solar absorption selectivity, which is strictly linked to both solar absorption and thermal emittance, can also be enhanced by the same procedure, therefore resulting in being beneficial for the application of sintered complex ceramics in solar applications [87]. The same technique was effectively extended to aluminum nitride-based absorbers [88], porous and dense tantalum carbide (TaC) [89], as well as tantalum diboride (TaB_2_) [81,90].

## 4. Non-Conventional Techniques for LIPSS Fabrication

A large extent of the results reported in the previous sections is the outcome of experiments performed by irradiating the surface of solid targets with a normal angle of incidence by a Gaussian beam profile of linearly polarized ultrashort laser pulses. However, during the last decade, this technique has been effectively modified in order to study either the fundamental origin of LIPSS or simply to obtain different and unique surface morphologies. Rohloff et al. [91] were the first to employ a process based on the irradiation of solid targets (i.e., fused silica) with sequences of temporally delayed and cross-polarized double pulses to survey the transient optical properties’ alterations of laser-excited materials, thus gaining new insight into the formation mechanism of LIPSS. Indeed, the work of Rohloff et al. was instrumental in confirming the significance of the promotion of laser-induced electrons in the conduction band and the consequent transient changes that are generated on a dielectric material during the LIPSS formation phase. The authors proposed that the first pulse locally induces a transient state in which the originally dielectric material can temporarily assume a metallic-like behavior characterized by the presence of dense electron plasma in the conduction band, while the second pulse has an amplification effect for sub-ps delays only [91]. The aforementioned transition occurs in silica on the timescale of 100–200 fs [92].

**Figure 7:**
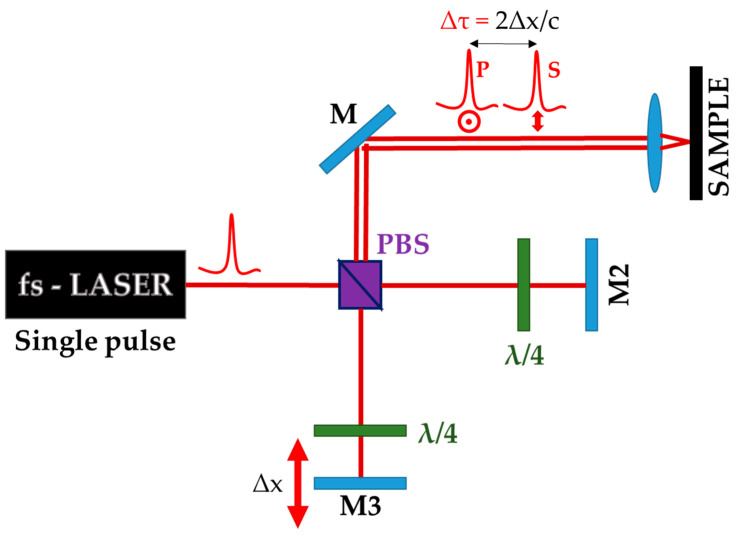
Schematic diagram of a Michelson-like interferometer configuration used for double-fs-pulse irradiations. PBS is a polarized beam splitter, M, M2 and M3 are mirrors, *Δτ* is the double-pulse delay induced by moving the M3 translation stage along the “*x*” axis (*Δx*).

In the optical setup used in this kind of experiment, a single pulse is transformed by a Michelson-like interferometer into two pulses with equal energy, and temporally delayed by *Δt*. The delay is adjusted by moving a linear translation stage that alters the optical path length, *Δx*, of one of the two reflection branches, as shown in Figure 7. The temporal delay could be varied with the aim of evaluating the ultrafast dynamics (up to 20 ps) of the processes occurring during LIPSS formation.

By irradiating different classes of materials (fused silica, silicon, titanium) with sequences of double-fs-pulses, Höhm et al. [92] confirmed that a single, universally extended LIPSS generation mechanism cannot be formulated since it can change significantly with the material’s properties onto which LIPSS formation takes place. Highly absorbing materials such as metals and narrow bandgap semiconductors provide an origin for LIPSS through plasmonic phenomena, but the same mechanism may not necessarily occur for dielectrics, which, as already defined in Section 2, are defined as plasmonically nonactive materials, although, transiently, they could behave as plasmonically active systems.

Time-resolved experiments based on the pump-and-probe methodology [93] were then conducted to further analyze the ultrafast dynamics occurring on fused silica. In this experiment, the probe beam impinges the sample at the center of the pump spot while its diffraction pattern, resulting from the interaction with the sample, is collected in transmission geometry. The experiment shows that a transient diffraction, caused by a periodic pattern at the LSFL wavelength, appears even before the surface of the sample is morphologically modified by the laser. This confirms that an ultrafast energy deposition is able to modulate the generation of electrons temporarily and spatially in the conduction band, transforming it into a strongly absorbent material.

Sequences of temporally delayed double pulses were also used to generate unique morphologies, such as complex two-dimensional LIPSS. In 2015, Cong et al. [94] reported the formation of two-dimensional dot-matrix subwavelength surface structures generated on molybdenum with a single irradiation step. Additionally, the authors demonstrated that the formation of the 2D-LIPSS was initiated and could be controlled by the transient interaction of the two pulses, which altered the ultrafast surface dynamics. In principle, by controlling the pulse delay, it is possible to transform the surface morphology as required.

In 2018, Qiao et al. [95] successfully produced periodic triangular arrays on tungsten using the same technique, and demonstrated that the surface treatment was able to greatly increase the spectral reflectivity of the material within the 700–2500 nm spectral range. Subsequent successful attempts were carried out on stainless steel [96,97] and cobalt [98], offering a flexible method for developing metasurfaces on metals.

**Figure 8:**
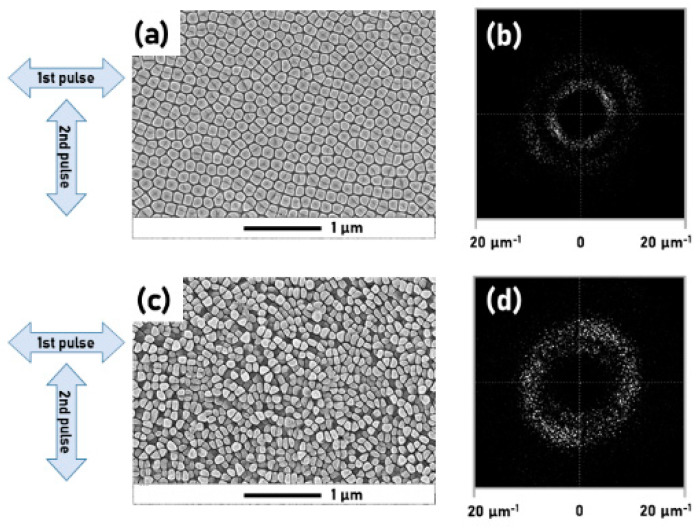
(**a**) SEM micrograph of 2D-LIPSS formed on the diamond surface after 40 double-laser pulses. (**b**) 2D−FFT of the SEM micrograph (**a**). (**c**) Formation of 2D−LIPSS on the diamond surface after 100 double-laser pulses. (**d**) 2D-FFT of the SEM micrograph (**c**). The arrows indicate the polarization directions of the first and second laser pulse. Reprinted with permission from Ref. [99]. Copyright © 2021 American Chemical Society.

However, it is our belief that this phenomenon is not solely restricted to metals, but it can be also obtained on WBS. In 2021, Mastellone et al. [99] reported the formation of 2D-LIPSS with a deep-subwavelength periodicity on diamond (*λ*/10), see Figure 8 (reprinted with permission from Ref. [99]).From this the limitations of conventional single-pulse-based techniques, which have been demonstrated to be effective only in inducing, on diamond substrates, irregular pseudoperiodic 2D-LIPSS [100,101] has been overcome. In Ref. [99] the authors indicated that it is possible to control the structure periodicities within the *λ*/10 ≤ *Λ* ≤ *λ*/4 range by adjusting the number of pulses impinging on the surface. 

Furthermore, it was demonstrated that the pulse delay is a fundamental fabrication parameter, since 2D-LIPSS on diamonds is only formed for *Δτ* ≤ 2 ps. The work was an additional confirmation of the model proposed by Dufft et al. for the formation of HSFL on WBS [37]. The model states that two criteria must be fulfilled for the HSFL formation: (a) Laser-excited electrons in the conduction band must exceed a specific threshold density to generate a surface-scattered electromagnetic wave, and (b) the incident beam and the surface-scattered electromagnetic wave must interfere.

In the context of employing laser beams with a profile different from the Gaussian one, an additional and innovative nano- and micro-patterning technique can be based on the use of optical vortex beams, namely laser beams in which polarization states are locally confined in complex spatial distributions or patterns that, in turn, allow the possibility of generating intricate surface structures. An optical vortex beam can be generated by letting the laser beam pass through a q-plate, i.e., an optical device able to perform spin-to-orbital conversion of the angular momentum of light. Vortex beams at an fs-scale can be exploited to fabricate new morphologies at micro- and nano-scales using an appropriate selection of beam polarization states, as it was demonstrated recently for silicon in Refs. [102,103], thus opening new paths in surface processing.

Finally, it is worthy to mention, another interesting technique for nano- and micro-patterning is Direct Laser Interference Patterning (DLIP), where the interference between two or more laser beams is employed to create unique periodic structures [104,105,106,107,108]. 

## 5. Conclusions

Surface nano- and micro-patterning by LIPSS is a challenging yet promising avenue for the development of a radically new class of surfaces for technological applications. It offers the possibility to modify and functionalize the surfaces of solids of any nature (metal, semiconductors and dielectrics) with a geometrical resolution lower than the one achievable through traditional optical systems restricted by the diffraction limit. 

As briefly reported, the fundamental mechanism that powers the formation of LIPSS is still under debate, so a definitive general consensus, and thus its integration at the industrial scale, is still in progress. The different models dealing with LIPSS have offered the opportunity to grasp the possible mechanism involved during its employment even for WBS and dielectric materials, which is mainly based on interferences among the incident laser beam and induced electromagnetic waves such as surface scattering and inhomogeneity nonradiative near-field patterns as well as transient SPPs’ activity due to the metallization-like process of the material’s surface. 

On this basis, it has been shown how the nonlinear multiphotonic excitation processes of carriers from the valence band to the conduction band of these materials (e.g., diamond) can provide structural and electronic structural changes, which can also result in electronic and compositional variations that can strongly affect the materials’ properties at their surface. It follows that together with the change of material topography ranging from LSFL to HSFL, which can strongly vary the material’s wettability [70,83,84] and optical properties [60], even functionalizations can be provided by carefully controlling all materials’ surface features as well as the laser beam and all the experimental parameters used (e.g., sample holder scanning speed, number of spatial effective incident pulses, material’s roughness and its preparation, laser incidence angle, surrounding environment). Thanks to LIPSS and relative processes, new perspectives in the fields of application of WBS and dielectrics can be added, such as, for instance, diamond for solar energy conversion [60], THz optical components [72] or new optoelectronic devices driven by intermediate band formation within the material’s bandgap, doping in the vicinity of surface defects or the assembling of new nanotemplate systems. 

Once the scalability, repeatability and controllability of preparation and processing methods of LIPSS has settled down, the correlation between the specific surface chemistry and topography will be conclusively established in the finished product. These will supply more drawings for applications of WBS and dielectric materials for which surface functionalization is one of the most important features (e.g., catalysts [109], nano and micro-electronics [110], bio-sensing [111], energy conversion [60,112], electrochemical and photoelectrochemical processes [71,113], elementary optical devices such as selective absorbers [80], THz optical components [72,114], etc.).

As it has been very briefly reported herein, different methods for varying LIPSS morphologies can be exploited (e.g., vortex beams, DLIP, pulse shaping); nevertheless, among these, the multiple fs-pulse irradiation LIPSS might offer more tunability in controlling the processes and, likely, the material’s surface properties by finely varying laser inter-pulse delays. It follows that well-defined tuning of the material’s electronic structure can be achieved by choosing the appropriate experimental parameters to be used, ensuring high homogeneities of the nanostructured surfaces so that the kinds and concentrations of vacancies induced, as well as the doping processes or building-block nanotemplates, can be exploited for further technological applications of WBS and dielectrics.

## Data Availability

Data sharing is not applicable to this article.
